# Entomologic investigation *of Plasmodium knowlesi* vectors in Kuala Lipis, Pahang, Malaysia

**DOI:** 10.1186/1475-2875-11-213

**Published:** 2012-06-22

**Authors:** Adela I Jiram, Indra Vythilingam, Yusuf M NoorAzian, Yusri M Yusof, Abdul H Azahari, Mun-Yik Fong

**Affiliations:** 1 Parasitology Unit, Infectious Diseases Research Centre, Institute for Medical Research, Jalan Pahang, 50588 Kuala Lumpur, Malaysia; 2 Parasitology Department, Faculty of Medicine, University of Malaya, 50603, Kuala Lumpur, Malaysia; 3 Entomology Unit, Infectious Diseases Research Centre, Institute for Medical Research, Jalan Pahang 50588, Kuala Lumpur, Malaysia

## Abstract

**Background:**

The first natural infection of *Plasmodium knowlesi* in humans was recorded in 1965 in peninsular Malaysia. Extensive research was then conducted and it was postulated that it was a rare incident and that simian malaria will not be easily transmitted to humans. However, at the turn of the 21st century, knowlesi malaria was prevalent throughout Southeast Asia and is life threatening. Thus, a longitudinal study was initiated to determine the vectors, their seasonal variation and preference to humans and macaques.

**Methods:**

Monthly mosquito collections were carried out in Kuala Lipis, Pahang, peninsular Malaysia, using human-landing collection and monkey-baited traps at ground and canopy levels. All mosquitoes were identified and all anopheline mosquitoes were dissected and the gut and gland examined for oocysts and sporozoites. Nested polymerase chain reaction (PCR) was conducted on positive samples, followed by sequencing of the *csp* gene.

**Results and discussion:**

*Anopheles cracens* was the predominant mosquito biting humans as well as the macaques. It comprised 63.2% of the total collection and was the only species positive for sporozoites of *P. knowlesi*. It was exophagic and did not enter houses. Besides *An. cracens*, *Anopheles kochi* was also found in the monkey-bait trap. Both species preferred to bite monkeys at ground level compared to canopy.

**Conclusion:**

*Anopheles cracens,* which belongs to the Dirus complex, Leucosphyrus subgroup, Leucosphyrus group of mosquitoes, has been confirmed to be the only vector for this site from Pahang during this study. It was the predominant mosquito at the study sites and with deforestation humans and villages are entering deeper in the forests, and nearer to the mosquitoes and macacques. The close association of humans with macaques and mosquitoes has led to zoonotic transmission of malaria.

## Background

Malaria still poses a public health problem in Malaysia despite continuous efforts to control the spread of the disease. Four species of *Plasmodium* were responsible for the spread of malaria in humans for a long time. However, currently the fifth species, *Plasmodium knowlesi*, which is also life threatening [1], has spread to many parts of Malaysia [2-4]. Besides Malaysia, knowlesi malaria has also been reported in countries in Southeast Asia. These include Thailand [5], Singapore [6], Philippines [7], Vietnam [8], Myanmar [9,10], Indonesia [11] and Cambodia [12]. This disease has also been reported in Europe and America by travellers who visited endemic sites in Southeast Asia, particularly Malaysia [13-17].

The first reported case of human knowlesi malaria in Malaysia was from Pahang. An American surveyor working in the jungles of Pahang contracted the disease [18]. Work was then carried out to determine the epidemiology of the disease and its vectors [19-23]. No further cases were detected in spite of screening more than 1,000 people in that area. No vectors were incriminated in that area during their study [21]. The main aim of the investigation was to determine if malaria was a zoonosis. Studies were also conducted in other areas of peninsular Malaysia. New species of simian malaria in macaques were described [24-26] and *Anopheles hackeri* was incriminated as the vector of *P. knowlesi*[23]. However, *An. hackeri* was found biting only macaques and was not attracted to humans. Thus, from the studies it was hypothesized that *P. knowlesi* will not be easily transmitted to humans and that the first case was an extremely rare event.

In peninsular Malaysia, malaria, which is transmitted largely by *Anopheles maculatus* is on the decline. However, cases of *P. knowlesi* are occurring in areas which have been free of malaria [3]. Early studies have incriminated the *Anopheles* Leucosphyrus group of mosquitoes as the vectors of simian malaria [21,27]. The epidemiology of knowlesi malaria is thus strongly linked to the Leucosphyrus group of mosquitoes. Recently *Anopheles latens* has been incriminated as a vector of *P. knowlesi* in Kapit, Sarawak Malaysian Borneo [28] and *Anopheles cracens* as the vector in Kuala Lipis Pahang, peninsular Malaysia [3]. The results of a longitudinal study to understand seasonal variation in two different ecotypes in Kuala Lipis is presented here.

## Methods

### Study sites for mosquito collection

The study was carried out in Kuala Lipis district in Pahang State, peninsular Malaysia. Three sites were selected for adult mosquito collection based on the presence of macaques and the occurrence of malaria cases. One was Serunai Mela Village [4° 7.0’N, 102° 11.9’E]. This area was at the edge of the forest and there were only two houses situated in that area, which were 300 m apart. Both houses were surrounded by trees and long-tailed macaques frequent the area. Towards the end of the study, parts of the forest were being cleared to build roads. The second site was a fruit orchard in Sungai Ular [4° 15.7’N, 102° 4.8’E]. This is a huge area with large trees on undulating land. Access to this area was controlled. More macaques were sighted in this area compared to the forest edge. The third site was in Sg Ular village [4° 12.673’ N, 101° 53.127’ E] where indoor and outdoor collections were carried out for three months. The houses in the villages were fairly close to each other and each house had its own yard planted with flowering plants and fruit trees.

### Mosquito collection

A 12-hour bare leg catch (BLC) [29] was carried out from August 2007 to August 2008 (with the exception of December 2007). Four nights of collections were carried out in each area every month by three men working in two shifts outdoors from 19:00 hours to 07:00 hours. The first shift was from 19:00 hours to 23:00 hours and the second from 23:01 hours to 07:00 hours. BLC was performed in the third site both indoors and outdoors by two men each from 19:00 to 23:00 hours. Indoor collection was discontinued after three months since no *Anopheles* mosquitoes were obtained indoors although they were biting outdoors and also partly due to shortage of manpower. It was carried out mainly to determine the presence of *An. cracens* and the behaviour, as cases were reported from the village (unpublished document). All volunteers who carried out mosquito collections were provided with doxycycline as the antimalarial prophylaxis.

### Monkey-baited trap (MBT)

In the forest (Serunai Mela), a study was conducted (simultaneously as BLC) to compare the mosquitoes attracted to human bait at ground level and monkey bait at ground level, and on platforms at 3 m and 6 m, in the forest canopy. The platforms were constructed as described by Wharton *et al.*[30]. In brief, the platforms were constructed among the branches of trees to a height of 6 m. Special metal cages measuring 90 cm x 90 cm x 90 cm and covered by chicken-wire were used to house the monkeys on the platform. In the first two months only one monkey (*Macaca fascicularis*) was placed in each cage but in subsequent months, two monkeys were kept in one cage. A mosquito net measuring 190 cm × 180 cm × 150 cm with an opening of about 40 cm on either end were used to cover the monkey cages on each platform. The traps were operated from 19:00 to 05:00 hours and were searched at 21:00, 00:00 and 05:00 hours. A collector, upon entering the net, closed the opening and collected all resting mosquitoes with the use of aspirators. Mosquitoes in the aspirator were then transferred to paper cups and were brought to the laboratory for identification and dissection.

### Mosquito identification and dissection

All mosquitoes were identified taxonomically in the field laboratory using a dissecting microscope. The keys of Reid [31] were used for the identification of *Anopheles* mosquitoes while the keys of Sallum [32] were used for the identification of Leucosphyrus group. Anophelines were dissected to extract the ovaries for the determination of parity and the midguts and salivary glands were examined for oocysts and sporozoites respectively. When sporozoites and oocysts were encountered, they were preserved in a 1.5 ml microcentrifuge tube (Axygen, USA) containing absolute ethanol. The tubes were labelled accordingly and brought to the laboratory for molecular studies.

### DNA extraction for salivary gland and oocyst

Prior to DNA extraction, ethanol used to preserve the oocysts and salivary glands were left to evaporate completely by placing the tubes in a Thermomixer (Eppendorf, Germany) set at 70°C. DNA was extracted using the Qiagen DNeasy Blood and Tissue Kit (Hilden, Germany), following the manufacturer’s recommendation.

### Nested polymerase chain reaction (PCR)

A nested polymerase chain reaction (PCR) assay described by Singh *et al.*[2,33] based on the *Plasmodium* sequence of the small subunit ribosomal RNA (SSUrRNA) was used to identify the species of malaria parasites found in the mosquito samples. The product from the first reaction (Nest 1) was used as the template for a second amplification (Nest 2). Positive controls for *Plasmodium falciparum, Plasmodium malariae, Plasmodium vivax* and *P. knowlesi* were included for all nested PCR species assays. A negative control from negative human blood was also included for every batch of the assays. The volume used for the Nest 1 reaction mixture was 50 μl. The PCR cocktail contained 1X reaction buffer (5X Green Go Taq Flexi Buffer, Promega Madison, USA), 3 mM MgCl_2_ (Promega), 200 mM of each deoxynucleoside triphosphate (Promega), 300 nM of each primer (rPLU1 and rPLU5) and 1.25 U of Go Taq DNA polymerase (Promega) and 5 μl of DNA template was used for each reaction. Nest 1 amplification conditions were as follows: initial denaturation at 94°C for 4 min; followed by 35 cycles of denaturation at 94°C for 30 sec; annealing at 55°C for 1 min; extension at 72°C for 1 min and a final extension at 72°C for 4 min. Two microliters (2 μl) of the Nest 1 PCR amplification products were used as the DNA template for each of the 20 μl Nest 2 amplification. Nest 2 reaction mixture contained 1X reaction buffer (5X Green Go Taq Flexi Buffer Promega), 2 mM MgCl_2_ (Promega,), 200 mM of each deoxynucleoside triphosphate (Promega,), 300 nM of each primers, and 0.5U of Go Taq DNA polymerase (Promega) and 2 μl of the Nest 1 PCR products were used as DNA templates. Nest 2 amplification conditions were identical to those of Nest 1 except that the annealing temperature was 58°C for the species-specific primers (rFAL 1 and 2, rMAL 1 and 2, rVIV 1 and 2), 60°C for *P. knowlesi* primers (Pmk8 and Pmk9) and 62°C for the genus-specific primers (rPLU 3 and rPLU4). All PCR reactions were carried out using thermal cycler (Techne TC 152 –Barloworld Sci Ltd, UK). Eight microliters (8 μl) of Nest 2 amplicons were loaded on a 2.5% agarose gel for 80 min at 80 volts using 1X TBE buffer. The gels were stained with ethidium bromide and were visualized under UV light.

### Sequencing of *plasmodium* circumsporozoite protein genes

Sequencing of the circumsporozoite protein (*csp*) genes were carried out on all four mosquito samples which were positive for *P. knowlesi* by nested PCR; isolates MO30SG, MO62SG, MO48SG and MO10MG. The *csp* genes were amplified using the protocol described [3]. The primers used were PKCSPF2 (5’TACAAGAACAAGATGARGAAC3’) and PKCSPR2 (5’TCAGCTACTTAATTGAATAATGC 3’). Phusion DNA polymerase (Finnzymes, Finland) was used for the PCR reaction. The size of the PCR product was approximately 1.2 kb and the amplicons from each isolate were excised from the agarose gel and purified using the QIAquick Gel Extraction Kit (Qiagen, Germany), following the manufacturer’s recommendation. The purified products were cloned into pCR Blunt vector (Invitrogen, Carlsbad, CA, USA) and transformed into TOP10 competent *Escherichia coli* cells (Invitrogen) by heat shock. At least 20 of transformants from each PCR were screened using the *csp* primers mentioned above. Amplification was done in 20 μl reaction mixtures containing 1X reaction buffer (5X Green Go Taq Flexi Buffer, Promega Madison, USA) 2 mM MgCl_2_ (Promega), 200 mM of each deoxynucleoside triphosphate (Promega), 300 nM of each primers and 0.5U Go Taq DNA polymerase (Promega,). PCR conditions were as follows: initial denaturation of 94°C for 10 min followed by 30 cycles of amplification at 94°C for 1 min, annealing at 53^o^C for 1 min, extension at 72°C for 1 min 20 sec, followed by a final extension step at 72°C for 5 min. Transformants with the correct band were grown in broth overnight. Plasmid DNA was extracted with the QIAprep Spin Miniprep Kit (Qiagen, Germany) following the manufacturer’s protocol. Ten microliters (10 μl) of the amplicons were digested with *Eco*R1 (Promega) and analysed by gel electrophoresis. Purified plasmids were sent to Solgent Co Ltd (Daejeon, South Korea) for sequencing to obtain the entire *csp* gene sequence of *P. knowlesi.* Two clones were sent for sequencing for each isolate with primers PKCSPF2 and PKCSPR2 [3].

### Analysis of sequence data

The *csp* genes of isolates were successfully amplified, cloned and sequenced. The analysis of *csp* was performed as previously described by [2]. For the *csp* gene, sequences of the 456 nucleotides that encodes non-repetitive N-terminal (first 195 nucleotides of coding sequence) and C-terminal (the last 261 nucleotides of the *csp* gene coding sequence) region of the protein were aligned. The nucleotide sequences of the *csp* genes were aligned by CLUSTAL W using Megalign (Lasergene, DNASTAR, USA). The sequences were compared to those obtained from the GenBank data base *P. knowlesi* from peninsular Malaysia (EU821335, EU821336, EU708437, EU687467, EU687468, EU687470), Sarawak Malaysian Borneo (AY327570, GU0025505), Thailand (JF923566); *P. coatneyi* (AY135360), *P. cynomolgi* (M15104), *P. simiovale* (U09765), *P. simium* (L05068), *P. inui* (GU002523), *P. fieldi* (GU002521), *P. vivax* (M34697), *P. malariae* (U09766), *P. falciparum* (K02194) and *P. vinckei lentum* (AF162331)]. Phylogenetic trees were performed by the neighbour-joining (NJ) method and was analysed using the Maximum Composite Likelihood method with 1,000 bootstrap replicates and was carried out using the MEGA version 4.0 software [34].

### Ethical clearance

This project was approved by the Institute for Medical Research & Ethical Committee, Ministry of Health, Malaysia.

## Results

### Species composition and spatial distribution of *Anopheles* species collected in different ecological sites

A total of 9,086 mosquitoes were obtained of which anophelines comprised 16.4%, while 83.6% were culicines. A total of 1,487 anophelines belonging to 14 species were collected within the 12-month period of mosquito collections from study sites as shown in Table 1. *Anopheles cracens* was the predominant species comprising 63.2% of the total collection of both bare leg catch and monkey-baited trap in the study sites. The second predominant anopheline species was *An. maculatus* (19.6%), followed by *An. hyrcanus* gr (4.4%) and *An. kochi* (3.6%).

**Table 1:** Anopheline mosquitoes collected from different collections sites in the district of Kuala Lipis, Pahang

***Anopheles*** **species**	**Number collected at**					**Total (%)**
	**Sg Ular**	**Kpg Serunai Mela**		Village house**		
	**(Fruit Orchard)**	(Forest)*				
		**BLC**	**MBT**	**Indoor**	**Outdoor**	
*An. aconitus*	3	0	0	0	21	24 (1.6)
*An. barbirostris gp*	5	12	14	0	6	37 (2.5)
*An. cracens*	648	179	73	0	40	940 (63.2)
*An. hyrcanus gp*	1	45	17	0	3	66 (4.4)
*An. kochi*	1	2	51	0	0	54 (3.6)
*An. leucosphyrus gp*	3	0	2	0	0	5 (0.3)
*An. maculatus*	203	61	3	0	25	292 (19.6)
*An. philippinensis*	7	6	0	0	4	17 (1.1)
*An. pujutensis*	1	0	1	0	0	2 (0.1)
*An. separatus*	3	6	2	0	0	11 (0.7)
*An. tessellatus*	5	8	7	0	5	25 (1.7)
*An. umbrosus*	0	1	1	0	0	2 (0.1)
*An. vagus*	0	1	0	0	0	1 (0.1)
*An. karwari*	2	8	1	0	0	11 (0.7)
**Total (%)**	**882 (59.3)**	**329 (22.1)**	**172 (11.6)**	**0**	**104 (7.0)**	**1487**

* MBT and BLC methods were used to collect mosquitoes at the forest while only BLC method was used to collect mosquitoes at other sites.

** Indoor and outdoor collections were only carried out for three months since no anophelines were obtained from indoor collections we discontinued due to manpower constraint.

### Species biting rate by ecological sites

Table 2 shows the biting rates for the four most predominant anthropophagic species and compares them with the different ecological sites. There were differences between both species composition and abundance in different locations. *Anopheles cracens* was the predominant species found abundantly in both the forest and the fruit orchard but was higher in the fruit orchard compared to the forest.

**Table 2:** Human biting rate (bites/man/night) of the 4 predominant anthropophagic species in different ecological sites

**Ecological sites**	**Person nights**	***Anopheles cracens***	***Anopheles maculatus***	***Anopheles philippinensis***	***Anopheles tessellatus***
Fruit Orchard	156	4.15	1.30	0.04	0.03
Forest	144	1.24	0.42	0.04	0.06
Outdoor	36	1.11	0.69	0.11	0.14

### Biting cycles

**Figure 1:**
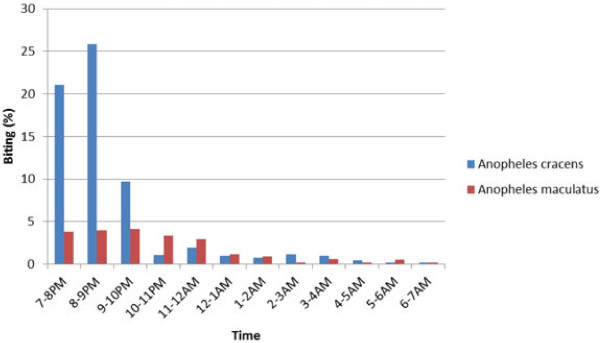
**Biting cycles of *Anopheles cracens *** **and *Anopheles maculatus *** **in study sites.**

*Anopheles cracens* are outdoor biters (exophagic). The other anophelines too did not enter houses. *Anopheles cracens* were early biters and come to bite man as early as 19:00 hours (Figure 1). The peak biting period was from 20:00 to 21:00 hours. Seventy four % of the *An. cracens* came to bite before 21:00 hours while 61% of *An. maculatus* was found biting after 21:00 hours. This was significantly different (P < 0.05).

### Seasonal changes in biting rate and parous rate

**Figure 2:**
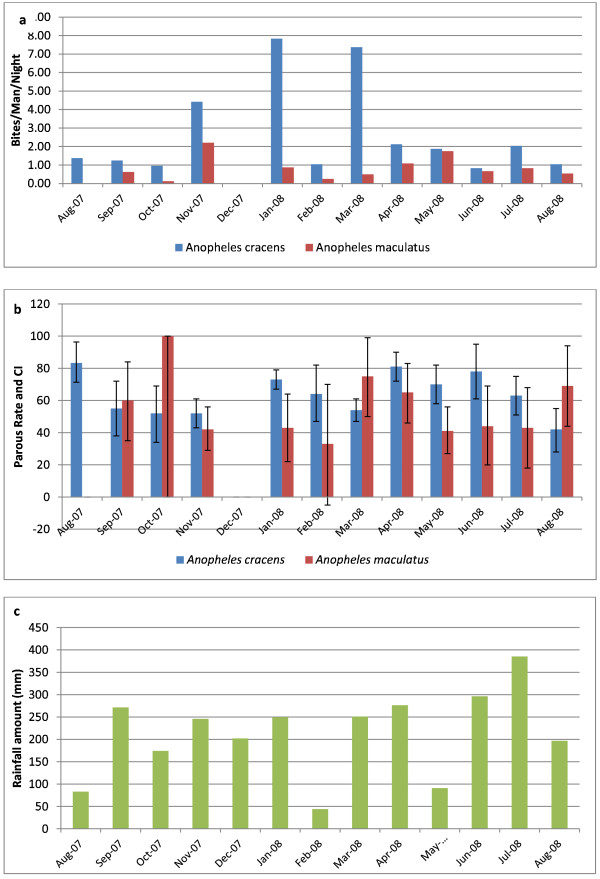
**a) Man biting rate of *Anopheles cracens *** **and *Anopheles maculatus *** **in study sites, b) Monthly parous rate and Confidence Interval for *Anopheles cracens *** **and *Anopheles maculatus *** **in study sites and c) Rainfall amount from August 2007 to August 2008 (Department of Orang Asli Affairs of Malaysia Meteorological Station, Kuala Lipis).**

Figure 2 shows the average bites/man/night and parous rate of *An. cracens* and *An. maculatus* in relation to rainfall. The nearest meteorological station was located at the Department for Aborigine Affairs in Kuala Lipis. Since meteorological data were available only at a central location and it has been observed that it rains in both places at the same time thus, the data for both areas have been combined. The biting peak of *An. cracens* was in November 2007, January and March 2008. Heavy rainfall followed by dry spell appears to be ideal for the breeding of *An. cracens*. However, there was no significant correlation between rainfall and abundance of *An. cracens* (using Pearsons correlation) (r = 0.12, P > 0.05) and *An. maculatus* (r = 0.0.25, P > 0.05). In most months the parous rate was more than 60%.

### Monkey-baited trap

A total of 172 anophelines were caught in the monkey-baited trap (Table 3). *Anopheles cracens* and *An. kochi* were the predominant mosquitoes found attracted to macaque monkeys. *Anopheles cracens* was more attracted to monkeys at ground level and 3 m from 19:00 to 00:00 hours. However, from 00:00 to 05:00 hours, *An. cracens* was more attracted to the monkeys at canopy level (6 m) compared to earlier collections at the same level. *Anopheles kochi* was predominantly biting at ground level. Based on the results from this study, the biting ratio of monkey to human of *An. cracens* is 1:2.6 and for *An. kochi* is 1:0.04

**Table 3:** **Numbers of** ***Anopheles cracens*** **and** ***Anopheles kochi*** **caught at different heights in relation to the time of collection in MBT in Mela village**

**HEIGHT**	**TIME**						**TOTAL (%)**
	1900-2100		2100-0000		0000-0500		
	*Anopheles cracens*	*Anopheles kochi*	*Anopheles cracens*	*Anopheles kochi*	*Anopheles cracens*	*Anopheles kochi*	
**Ground (0 m)**	15	25	12	6	10	11	79 (63.7)
**3 m**	9	2	8	2	5	1	27 (21.8)
**6 m**	0	0	4	4	10	0	18 (14.5)
**TOTAL (%)**	24	27	24	12	25	12	124

### Infection rates in *anopheles*

Three *An. cracens* were found positive for sporozoites of which two were from human bait collection and one from monkey bait collection. One was positive for oocyst. In both study sites, sporozoite-infected *An. cracens* were obtained. Only one *An. cracens* had both oocyst and sporozoites. Three of the sporozoite infections were *P*. *knowlesi.* The risk of infection was high during the particular month (November 2007 and January 2008) when infected mosquitoes were found as shown in Table 4.

**Table 4:** **Man-biting rate, sporozoite rate, entomological inoculation rate, estimated mean inoculation per month and risk of receiving infection of** ***Plasmodium knowlesi*** **from** ***Anopheles cracens*** **in study areas**

**Species**	**Study Site**	**Months (Sporozoite)**	**Man-biting rate (ma)**	**Sporozoite rate *s* (95% CI)**	**Entomological inoculation rate (EIR)**	Risk*
*Anopheles cracens*	Fruit Orchard	January 2008	13.5	0.60 (0.52- 0.68)	0.08	0.92
	Forest	November 2007	2.8	2.90 (2.11-3.30)	0.08	0.91

*Risk calculated using the formula 1-℮ ^-inoculation/month^.

For the particular month sporozoites were present.

### Parous rate, probability of survival, life expectancy and vectorial capacity

The parous rates of *An. cracens* and the confidence interval are shown in Table 5. More than 60% of *An. cracens* were parous,and 31% of these would be expected to live the 10 days necessary for the *P. knowlesi* sporozoites to be formed. Those would have a further life expectancy of 8.6 days. However, the vectorial capacity was higher in fruit orchard compared to the forest due to higher biting rate. Vectorial capacity was calculated according to Garrett-Jones and Shidrawi [35].

**Table 5:** **Parous rate, probability of daily survival, life expectancy (days) and vectorial capacity of** ***Anopheles cracens*** **in study areas**

	**Fruit Orchard**	**Forest**
Parous rate (95% CI)	65.7 (62.0-69.4)	71.5 (65.9-77.1)
Probability of daily survival - p	0.87	0.89
p10 (%)	25	31
Life Expectancy p10/-log_*e*_p (days) (1/-ln p)	7.2	8.6
Vectorial Capacity	2.46	1.09

### Analysis of the *csp* genes sequencing

**Figure 3:**
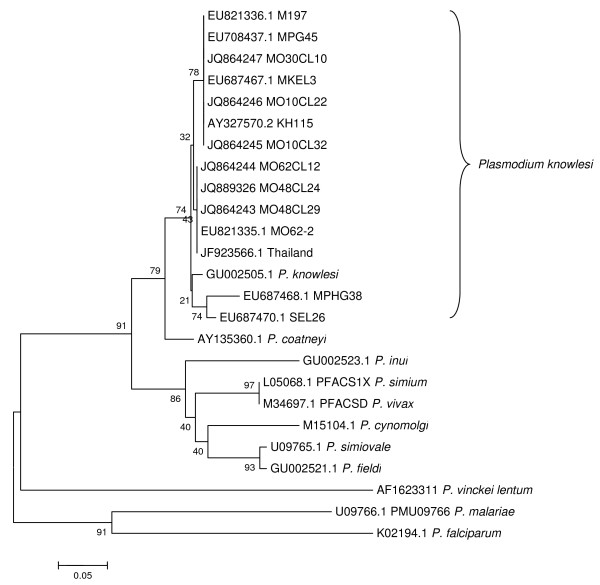
**Phylogenetic tree based on csp sequences of Plasmodium spp.** JQ864243, JQ864244, JQ864245, JQ864246 JQ864247 and JQ889326 are *P. knowlesi* csp sequences obtained in this study. The tree is constructed using neighbour joining method MEGA4 [34]. The percentage of replicate trees in which the associated isolates cluster together in the bootstrap test (1,000 replicates) are shown next to the branches.

The *csp* genes of malaria parasites, from the *P. knowlesi* isolates were successfully amplified, cloned and sequenced. All sequences for *csp* clones from four mosquito isolates were aligned and compared with four human malaria and all simian malaria species. The target size for PCR product ranges from 1,028 to 1,200 bp. Nucleotide sequences obtained were compared with other reference sequences by using the nucleotide BLAST to find significant matches. The sequences from the mosquito samples [JQ864243, JQ864244, JQ864245, JQ864246 JQ864247 and JQ889326] showed similarity with the monkey and human samples from the east coast region of peninsular Malaysia and Sarawak (Figure 3).

## Discussion

Knowlesi malaria is an emerging zoonosis of public health importance in Malaysia. Owing to Malaysia’s rapid development, tropical climate, presence of vectors and long-tailed macaques, timely and effective disease control is required to prevent the spread of the disease. Since the emergence of knowlesi malaria in the country in 2004 [2], entomological investigations have been carried out to elucidate the vectors. *Anopheles latens* and *An. cracens* have been incriminated as the vectors in Kapit, Sarawak [28] and Kuala Lipis, Pahang [3] respectively. Both these species are members of the An. leucosphyrus subgroup of mosquitoes known to be natural vectors of simian malaria. Species belonging to this group are also important vectors for human malaria and are distributed in the South and Southeast Asia region [36-38]*. Anopheles latens* is also a predominant vector of human malaria in Sarawak and has widespread distribution there [39,40]. However, *An. cracens* has limited distribution in the northern state of Perlis [19], in the east coast state of Terengganu [32,41] and currently in Pahang. It was also reported from southern (peninsular) Thailand (Chumphon, Phangnga, Phattalung) [42], and Indonesia (Aceh, Sabang, Sumatra) [32]. *Anopheles cracens,* was found to be an important vector not only for human malaria, but was also positive for *P. inui* and *P. cynomolgi*[19]*.* This mosquito was found to be attracted to monkeys at canopy and humans at ground level [43]. However, the current study has shown that *An*. *cracens* is biting macaques more at ground level than at canopy. The propensity of *An. cracens* to bite monkeys at ground level or canopy, and humans, demonstrates the importance of this species in the transmission of knowlesi malaria. Due to changes in the ecosystem, the behaviour of the mosquito also seems to change. Although extensive vector studies have been carried out in Pahang in the 1980s and 1990s by researchers from the Institute for Medical Research, (Kuala Lumpur, Malaysia) [29,44,45] there have been no previous reports of *An. cracens* there. Earlier studies by Wharton *et al.* in other parts of Malaysia also show no reports of this species [22].

Studies carried out by Baimai [42] in Thailand showed that *An. cracens was* an anthropophilic species and peak biting activity was from 19:00 to 21:00 hours. Thus, it seems that *An. cracens* has not changed its biting activity since similar times has been demonstrated in this study. In addition, *An. cracens* was known to enter shelter but rarely rested on walls and readily exited after a bloodmeal. *Anopheles cracens* was an important vector of human malaria because it was involved in the human *Plasmodium* transmission in areas where houses were close to the jungle [46]. After the 1960s very little is known about *An. cracens* in Malaysia*.* The distribution of *An. cracens* in peninsular Malaysia remains unknown, although cases of knowlesi malaria are reported from all states in Malaysia.

Despite high prevalence of simian malaria in macaques in Kuala Lipis [3], the infected mosquitoes obtained from the study were low. Thus, there is a possibility that other species besides *An. cracens* are involved in the transmission of simian malaria at least among macaques. Transect studies through the forest will determine the other species involved in transmission. Studies carried out in Kapit, Sarawak where a large number of knowlesi malaria cases were reported from that area, *An. latens* was incriminated as the vector and the monkey to human biting ratio was 1:1.3 [47]. In comparison to that, the monkey to human biting ratio for *An. cracens* was 1:2. From this study, it was observed that *An. cracens* is attracted to both monkey and human. However, it prefers to bite humans compared to monkeys. This could possibly be the reason why there was fewer knowlesi malaria cases in peninsular Malaysia compared to Malaysian Borneo. The frequency of a man-monkey-mosquito natural cycle transmission is dependent upon the various hosts in the area of natural transmission. *Anopheles hackeri* was incriminated as the natural vector of *P. knowlesi* among the monkey population in peninsular Malaysia after the discovery of the first human case of *P. knowlesi* in Pahang [23]. However, *An. hackeri* is not attracted to humans and is zoophagic. Thus, at that time it was concluded that *P. knowlesi* would not be easily transmitted to humans due to the nature of that mosquito. Thus, it is evident that more studies on vectors are needed throughout Malaysia in order to understand the dynamics of simian malaria being transmitted to humans.

Laboratory studies have shown both *An. kochi* and *An. maculatus* are susceptible to simian malaria parasites especially to *P. cynomolgi*[21]. However, the numbers of *An. maculatus* coming to the monkey-bait trap was very scarce and this could be a reason why *An. maculatus* has not been found with natural infection of simian malaria. As for *An. kochi,* it was found predominantly at ground level and not at canopy level and this could be one of the reasons why it was not positive for sporozoites. In nature the macaques roost on trees at night and thus mosquitoes biting at canopy level will be able to pick up infection.

Although knowlesi malaria has been reported from many countries in Southeast Asia, studies on vectors in relation to knowlesi malaria are lacking. Besides Malaysia, studies in Vietnam have incriminated *Anopheles dirus* to be the vector of *P. knowlesi*[48,49]. In Vietnam *An. dirus* has been found positive with mixed infection of human and simian malaria sporozoites. However, cases of knowlesi malaria in Vietnam was cryptic and only a few cases have been reported [8], while in Malaysia, mixed infection of human and simian malaria occurs [2,3] but no mixed infection was found in *An. cracens*. This leads to further gaps in our knowledge with regard to human to human transmission or monkey to human transmission. In the case of Malaysia, deforestation has certainly disrupted the ecosystem and the forest mosquitoes that were found in the northern part only of peninsular Malaysia is now reported in the east coast of the peninsular and were found in the edge of the forest and in villages surrounding the forest. How much further they have spread remains unknown. It is also difficult to decipher why the numbers of *An. cracens* were more in the fruit orchard compared to the forest edge. With deforestation this species of mosquito is still able to colonise the plantations and the forest edge. It seems similar to a report in Thailand where by *An. dirus* a forest mosquito colonised plantations when deforestation took place [50]. Since cases of knowlesi malaria have been reported from all states of Malaysia, vectors must be present everywhere. However, a species may play a primary role in an area and a secondary role elsewhere. Thus, vector control activities will be hampered unless vectors are elucidated throughout the country. In order to eliminate malaria in Malaysia it is important to study the vectors in various ecological zones so as to design an effective control programme. From this study it is also clear that current vector control tools being used in the malaria control programme will not be effective to reduce vector population as these mosquitoes are exophagic and early biters. Extensive studies should also be carried out on village population to determine the prevalence of knowlesi malaria in the country. Besides microscopy, molecular techniques should be used in order to determine the species of malaria. Only then can proper strategies be instituted to control malaria and work towards its elimination.

Molecular techniques are very useful in identifying the infection, in describing the epidemiology, and in characterizing mixed infections, which are otherwise under reported. Thus, with the improvement in molecular diagnostics methods, one is now able to distinguish between *P. knowlesi* and *P. malariae.* However, the levels of the problem strongly rely on the cohabitation of the monkeys, humans and the presence of the vectors, which are simio-anthropophagic and exophilic. The frequency of man-monkey-mosquito natural cycle transmission is dependent upon the various hosts in the area of natural transmission.

## Conclusion

This study has confirmed that *An. cracens*, has the highest vectorial capacity among all the anophelines collected from Kuala Lipis, Pahang. The behavioural studies revealed that *An. cracens* is simio-anthropophagic, acrodendrophilic towards the later part of the night and is the natural vector of *P. knowlesi* and other simian malaria parasites. Identification of vectors involved in the transmission of *P. knowlesi* to humans has been established and this should lead to the appropriate control strategies for elimination of malaria as the indoor residual spraying and insecticide-treated bed nets are insufficient to control the vectors of simian malaria due to the strong exophilic and exophagic nature of the vector. Thus, there is an urgency for similar studies to be carried out in other states of Malaysia as cases of knowlesi malaria are reported throughout the country and the vectors responsible remain unknown in most states of Malaysia.

## Competing interests

The authors declare that they have no competing interests.

## Authors’ contributions

IV conceived the study. AIJ, IV and FMY were responsible for the preparation of the manuscript. IV, AIJ, NAY, YY and AAH were responsible for field collection, supervision, identification and processing of mosquitoes. AIJ and IV were responsible for the molecular work. AIJ, FMY and IV analysed sequence data. All authors have read and approved the manuscript.
